# Meat Demand Monitor during COVID-19

**DOI:** 10.3390/ani11041040

**Published:** 2021-04-07

**Authors:** Glynn T. Tonsor, Jayson L. Lusk, Shauna L. Tonsor

**Affiliations:** 1 Dept. of Agricultural Economics, Kansas State University, Manhattan, KS 656506, USA; 2 Dept. of Agricultural Economics, Purdue University, West Lafayette, IN 47907, USA; jlusk@purdue.edu; 3 Elevation Economics, LLC, 4255 Christi Ln., St. George, KS 66535, USA; elevationecon@gmail.com

**Keywords:** beef, consumer behavior, COVID-19, meat demand, pork, survey

## Abstract

**Simple Summary:**

Meat products represent a core aspect of the US food supply chain and faced multiple disruptions in the COVID-19 pandemic. Available data are often aggregated or lagged in availability, thus limiting insights into consumer behavior. Using household-level data from continuous consumer tracking in the Meat Demand Monitor project, we extend understanding of consumer behavior during the pandemic. This provides a timely example of the improved insights that follow from refined and ongoing data collection to generate valuable information.

**Abstract:**

Meat products represent a significant share of US consumer food expenditures. The COVID-19 pandemic directly impacted both demand and supply of US beef and pork products for a prolonged period, resulting in a myriad of economic impacts. The complex disruptions create significant challenges in isolating and inferring consumer-demand changes from lagged secondary data. Thus, we turn to novel household-level data from a continuous consumer tracking survey, the Meat Demand Monitor, launched in February 2020, just before the US pandemic. We find diverse impacts across US households related to “hoarding” behavior and financial confidence over the course of the pandemic. Combined, these insights extend our understanding of pandemic impacts on US consumers and provide a timely example of knowledge enabled by ongoing and targeted household-level data collection and analysis.

## 1. Introduction

During the COVID-19 pandemic in the US, the term “essential” was widely used in relation to the food sector. The meat category was a high-profile sector of the US food supply chain as the pandemic brought consumption, production, and availability of meat into the national spotlight. This elevated interest reflects meat being a core, even essential, component of diets for most US residents. In 2019, consumers spent over $1 trillion on food and beverages for off-premise (away from home) consumption with meat representing the largest (17%) category at about $176 billion [1]. Beef (27%), pork (20%), and poultry (33%) comprise the majority of these meat expenditures. Going further to consider both at-home and away-from-home activity, total red meat and poultry consumption in 2019 was 224 lbs per person—the highest on record going back to 1960 [2]. These data suggest that the average resident consumed 0.61 lbs of meat each day in 2019. Given the prevalence of meat in US diets and the combined economic relevance of substantial consumer expenditures, it is important to understand impacts COVID-19 had on US meat consumers.

Given its central role as the predominant protein entrée for the majority of US consumers, and the associated magnitude of economic importance for US agriculture, it is not surprising to find decades of agricultural economics research on the meat industry. Studies on consumer demand for meat include the following: meta-analysis of own-price elasticity of meat demand [3], separability of meat product categories [4], structural change in meat demand [5], impact of food safety events on meat demand [6], consumer welfare impact of possible bans on antibiotic use in pork production [7], economic value of enhancing beef tenderness [8], role of government in nudging consumers toward “optimal meat consumption” [9], and assessing how cattle producers are impacted by changes in consumer beef demand [10]. Clearly, there is high interest in this consumer expenditure category, emphasizing the need to understand COVID-19 impacts.

Studies of meat demand have entailed an ongoing evolution of data and methods employed. In past decades, it was common for analyses to largely rely on highly aggregated, government-sourced data. Over time, the nature and detail of available data has evolved, and with it an increase in more refined, household-level analyses collected on a continued basis. Lusk (2017) makes multiple points specific to the meat demand and how refined insights can be obtained by use of ongoing online surveys and choice experiments [11].

In that spirit, it is important to appreciate most existing datasets are not well suited to provide timely and refined insights on the array of economic issues presented by COVID-19. While monthly retail meat prices and quarterly per capita disappearance (a predominant proxy for consumption) estimates reported by the USDA (United States Dept. of Agriculture) provide useful information, this information is released with significant time delay and aggregation over product, households, and market-channels. Related context is provided by Tonsor (2019) and Bekkerman, Brester, and Tonsor (2019) in how publically available, aggregate data is used to support US meat demand indices that are available 6 weeks or more after conclusion of a calendar month [12,13].

This article utilizes data from a recently launched consumer tracking survey, the Meat Demand Monitor, which began in February of 2020 just weeks before the pandemic changed the lives of US residents. These data permit unique insights into understanding the evolving impacts of COVID-19 for US meat consumers. The survey also allows us to measure changes over time, including behavioral changes such as at-home “hoarding” and shifts in consumers’ financial conditions. Such changes were particularly stark during COVID-19. For example, the University of Michigan Index of Consumer Sentiment had a 19% drop from February to March 2020, the biggest drop in consumer sentiment between consecutive releases since 1952 [14]. Aggregate production disruptions largely improved by July [15], accordingly we focus on Meat Demand Monitor insights over the February–July period where consumers were experiencing largest changes.

The next section provides some background on the timeline of meat sector disruptions before discussing the tracking survey in more detail. We then present a variety of results related to consumer behavior and preferences during the pandemic including insights into reported hoarding behavior, stated responses to price changes, and analysis of trends in preferences for meat at home and away from home. The last section concludes and provides related guidance encouraging future data and information collection efforts.

### Timeline of Meat Sector Disruptions

Perhaps no segment of the US food supply chain during COVID-19 has experienced the combination of demand shocks, supply disruptions, internal industry strife, and calls for government involvement than the meat-livestock sector [15]. In March, as US states began introducing a myriad of stay-at-home orders and many employees began to work from home, a large aggregate shift of food occurred from away-from-home food consumption to at-home consumption. In the meat sector, this is important as many products are typically targeted for one particular market channel. Some products such as bacon and high-quality beef steaks are typically consumed more away-from home while others were already heavily consumed at-home such as ground beef and pork chops [16]. Combined, this led to abrupt shocks across meat categories.

In April, supply-side challenges developed as livestock packers and meat processors struggled to sustain production in light of worker illnesses and plant closures [17]. The traditional dependence on labor now impacted by a human pandemic, resulted in beef and pork production being down nearly 40% in the final week of April compared to the same week in 2019. Not surprisingly, the preceding push by consumers to build at-home inventories coupled with meat production disruption and subsequent price spikes led to wide discussion throughout major media outlets in April and May [18,19].

## 2. Materials and Methods

In February of 2020, with joint funding support from the pork and beef checkoffs, a new project called the Meat Demand Monitor (MDM) was initiated. The MDM is an extension of the Food Demand Survey which is mainly focused on US meat demand in both retail and food service outlets [11,20]. 

On a monthly basis, over 2000 US residents enrolled in an online panel maintained by Dynata (formerly Survey Sampling International) are surveyed. Each month a different set of respondents participate. Monthly data are collected with intent to be nationally representative of the US population by age, education, gender, geographic region, household income, and race-ethnicity. Additional details, including links to base monthly and special multi-month reports, raw data, and survey instrument files, are available on Kansas State University’s AgManager.info website: https://www.agmanager.info/livestock-meat/meat-demand/monthly-meat-demand-monitor-survey-data (accessed on 5 April 2021). Estimates reported here, and in all project outputs, also reflect sample weighting using the RAKING macro in SAS. In particular, the sample is weighted using Census estimates to reflect the national population in age, education, gender, geographic region, income, and race-ethnicity.

Beyond targeting nationally representation, data quality filters are used to mitigate impact from undesirable responses. The specific filters applied are detailed in Tonsor (2020) and result in retaining responses from participants who are involved with grocery shopping for the household, who stated ages between 18 and 120 years old, who passed a simple speeder-trap question, and who indicated in the survey’s final question they answered all questions to the best of their ability [21].

The broader survey contains questions documenting consumption patterns by meal and location the prior day, relative importance of various factors in protein purchasing decisions, choice experiment questions for monitoring meat demand, and an “ad hoc” section enabling timely assessment of hot topics. In many cases, presented answers or sub-items of a question are randomized in the order of presentation to mitigate presentation order effects on conclusions. Several components from over 12,000 respondents during February–July 2020 are used in this paper and underlying methods are discussed further in the remaining paragraphs of this section. 

**Figure 1:**
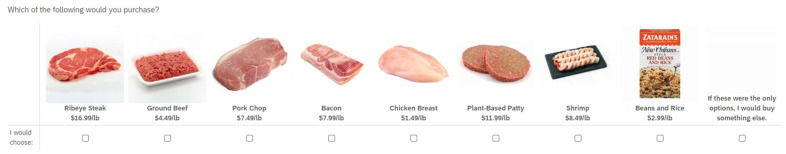
Example choice experiment question for retail setting.

**Figure 2:**
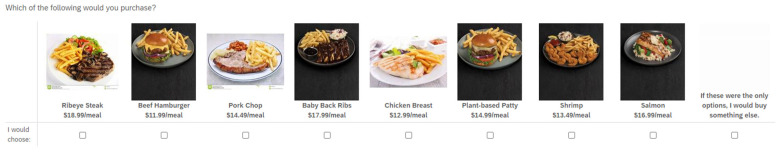
Example choice experiment question for food-service setting.

A central aspect of the MDM project is collecting information for meat demand in retail (grocery) and food service (restaurant) sectors. Given the growth over time in prevalence of food service consumption, the need for expanded insight on meat demand specific to each domestic market channel is clear. Survey participants are randomly allocated to either a survey containing a retail sector framed choice experiment sequence or a food service sector sequence. In each choice experiment, each participant answers nine questions with examples presented in Figure 1 and Figure 2.

Before the retail questions, the survey instructions presented were as follows: “Imagine you are at the grocery store buying the ingredients to prepare a meal for you or your household. Each product would be boneless and uncooked for you to prepare at home as desired. For each of the following 9 questions, please indicate which you would most likely buy. The only difference across these 9 questions is the price ($/lb) of each option”. Similarly, the following was presented to those subsequently receiving food-service questions: “Imagine you are at your local restaurant for dinner. For each of the following 9 questions, please indicate which main entrée you would most likely select for your meal. Each product would be the dinner meal’s main entree, would be prepared as you desire, and served with two side dishes of your choosing. The only difference across these 9 questions is the meal price associated with each main entrée option”.

Each question presented nine options comprised of eight protein options and one “no purchase” or “opt out” alternative. Three price levels for each option are included following the same design as Lusk (2017) [11]. Mid-point price levels were selected to reasonably represent early December 2019 prices during the final project design process. Price increases and decreases of $2.50 were then used resulting in a range of $5 for each option across the entire set of choices. The orthogonal fractional design comprised of 27 questions. To mitigate respondent burden, these 27 questions were randomly blocked into sets of nine and each person was randomly assigned to one block.

The resulting choice data is analyzed each month, separately by market channel yielding retail and food service sector insights. Consistent with typical application of a random utility model, it is presumed that consumer *i* derives utility from choice option *j* [22]:

(1) $$U_{ij}=V_{ij}+\varepsilon_{ij}.$$

The conventional multinomial logit model is applied with the deterministic portion of utility specified as follows:

(2) $$V_{ij}=\beta_{j}+\alpha p_{j}$$

where $p_{j}$ is the price of option *j*, $\alpha_{}$ is the marginal utility of price changes, and $\beta_{j}$ is an alternative specific constant representing the utility of choice option j relative to the opt out, no purchase option. Upon model estimation, willingness to pay for each choice option relative to “no purchase” is easily recovered by taking the ratio of the alternative specific constant to the price effect.

Each month, additional ad hoc questions are also included to gather timely insights. Coinciding with the Meat Demand Monitor launching in February of 2020, to-date most of the ad-hoc questions have focused on pandemic issues relevant to the meat demand. In May, June, and July, the MDM survey included questions providing insight into at-home supplies of meat by asking the following: “How would you describe the amount of meat your household currently has on-hand at home (e.g., in refrigerator or freezer)?” with available answers being “more meat on-hand than normal”, “same amount as normal”, and “less meat on-hand than normal”.

To help assess possible impact of retail price increases that were uncertain yet of elevated interest early in the pandemic given meat production disruptions, in May survey respondents were randomly allocated to receive one of four multiple choice questions. The presented question framed around a 25% beef-price increase was as follows: “Suppose tomorrow you are shopping for your favorite beef product and it is available for purchase at a price 25% higher than last time you shopped. What best describes your decision?” Five answer options were presented including “I would buy my favorite beef product, at the same quantity as planned”, “I would buy my favorite beef product, but at a lower quantity than planned”, “I would alternatively buy a pork product”, “I would alternatively buy a chicken product”, and “I would not buy a beef, pork, or chicken product”. Three parallel questions were framed around a 50% price increase or alternatively for pork price increases were also asked.

Consistent with the surge in economic uncertainty and the historic role consumer confidence has in purchasing of many goods, beginning in April sentiment questions from the long-running University of Michigan, Consumer Sentiment project were added. Specifically, respondents are asked “Would you say that you (and your family living there) are better off or worse off financially than you were a year ago?” where answers provided were “Better Now”, “Same”, “Worse Now”, or “Don’t Know”.

As a final data and methods point, additional Meat Demand Monitor project methodology details are detailed in Tonsor (2020) [21]. In the Results section we summarize base data trends from the MDM project to gain insight regarding consumer meat purchasing behavior, sensitivity to price changes, and mean willingness-to-pay for meat items in retail and food service channels.

## 3. Results

### 3.1. Consumer Behavior Changes—Should We Call it Hoarding?

Given widespread images of empty retail shelves and “hoarding” commentary in the media [19,23] when federally inspected beef and pork production was substantially reduced in late April, a key question facing economists was how consumers would respond. Central to this question was what at-home inventories consumers held and to what extent meat purchasing behavior in March and early April enabled consumers to smooth their meat consumption given bottleneck production challenges that subsequently disrupted the flow of products available to consumers. In May, June, and July, the MDM survey included questions providing insight into this issue. When asked “How would you describe the amount of meat your household currently has on-hand at home (e.g., in refrigerator or freezer)?” Moreover, 61%, 66%, and 65% indicated “same amount as normal” in May, June, and July, respectively. Meanwhile in May, June, and July, 13%, 12%, and 14%, respectively, indicated “less meat on-hand than normal”. The remaining 26%, 22%, and 21% in May, June, and July, respectively, indicate they have “more meat on-hand than normal”.

Thus, the majority of consumers held normal stocks, and those who built-up larger stocks outnumbered those with smaller stocks than normal. On balance, this indicates meat consumption was likely smoothed, in part, by proactive consumer purchases before mid-April, when the industry began having significant production disruptions (e.g., note 26% in May had larger stocks versus 21% in July). The extent extra meat was purchased only to be wasted (e.g., discarded rather than consumed) was likely reduced, along with the disruption in beef and pork production in April and May. Given the benefit of hindsight, at this point, perhaps “preparing to smooth consumption” would more accurately describe the March–early April “empty grocery shelves” situation. Thus, while behavior was often interpreted as “irrational”, there were, in fact, many reasons motivating consumers to move inventory from grocery stores into their homes [24].

Beginning in May, respondents were also asked if the volume and type of meat options available was normal and consistent with the past. In May, 54% reported meat options were normal and consistent. This proceeded to rise to 58% in June and 65% in July, reflecting the recovery in federally inspected meat production which occurred.

### 3.2. Stated Price Sensitivity

**Figure 3:**
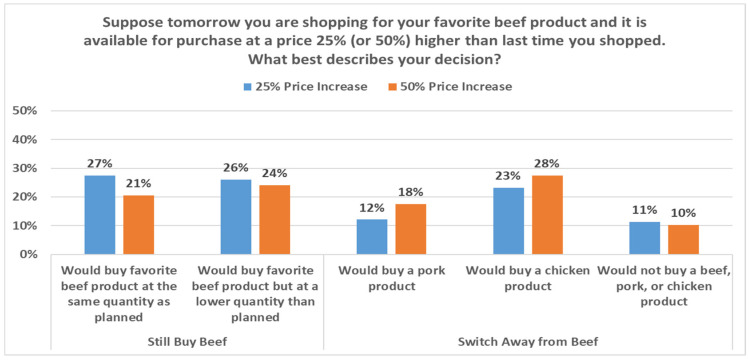
Response to beef-product price increase, May 2020.

**Figure 4:**
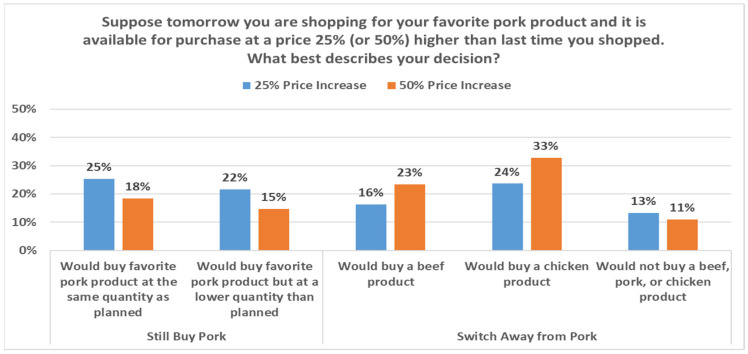
Response to pork-product price increase, May 2020.

Beyond questions about availability, much uncertainty surrounded retail meat prices as April concluded. Accordingly, economists faced questions around how consumers would respond to elevated retail meat prices resulting from the reductions in supply. To help assess possible impact of retail price increases given production disruptions, May survey respondents were randomly allocated to receive one of four multiple choice questions. The question framed around a 25% beef price increase was as follows: “Suppose tomorrow you are shopping for your favorite beef product and it is available for purchase at a price 25% higher than last time you shopped. What best describes your decision?” Parallel questions for a 50% price increase and alternatively for pork price increases were also asked. Figure 3 and Figure 4 summarize responses to these four questions.

As expected, larger price increases result in a larger number of respondents reducing the volume they stated they would purchase and substituting out of the more expensive meat category. Consumers switching from their preferred cut largely indicated a move toward chicken, which is noteworthy given that the poultry industry has experienced fewer production disruptions, to date, in the pandemic [15].

We can leverage differences in responses from the presented price increases to gain insight on cross-price effects in a new way that may augment more traditional demand-system approaches and even choice experiment approaches such as the one we present below. For instance, consider the impact on pork demand from beef price increases. The larger share (18% vs. 12%) indicating they would purchase pork following a 50% rather than 25% increase in the price of their favorite beef item implies a cross-price elasticity of +0.22. The differences in responses to 25% and 50% responses are used to help mitigate any external effects of the question. Here, there is an increase of 5.49% (17.58–12.09%) in the share selecting pork, following a 50% beef price increase, compared to a 25% price increase. Presuming linear changes, this yields a 0.2196% cross-price elasticity estimate (5.49%/25%). For each 1% increase in beef price, these data suggest a 0.22% increase in pork demand. Similar calculations suggest that, for each 1% increase in beef price, the chicken demand increases by 0.17%, and for each 1% increase in pork price, the beef demand increases by 0.28% and chicken demand increases by 0.37%. Collectively, this suggests consumers substitute away from pork more quickly than beef when faced with higher prices. This is consistent with years of research indicating that beef demand is more inelastic than pork demand [25,26].

It is also worth highlighting the small estimates (13% or fewer in the presented figures) for those indicating they would purchase something besides beef, pork, and chicken. This rather small exit provides indirect support for other research which may impose meat-separable assumptions [27]. That is, this suggests, indeed, the bulk of adjustment by US meat consumers may be within the meat-protein space.

### 3.3. Meat Preferences over Time

By chance, the MDM project launching in February of 2020, corresponds well with the onset of the US COVID-19 experience. Accordingly, it is helpful to summarize patterns in market-channel-specific demand.

Table 1 and Table 2 show the mean willingness-to-pay (WTP) by month for the February–July period, for retail and food service, respectively, resulting from multinomial logit models fit to the choice experiment data. As noted by a reviewer, there are differences between estimating mean WTP values and full demand curves [20]. Here we focus on differences in mean WTP over time and across cohorts; yet we highlight that future wok could extend this analysis to map out demand curves. 

The spike in retail demand ($/lb) and decline in food-service demand ($/meal) in April is consistent with stay-at-home orders and broader shifts in consumer purchasing activity. 

To further appreciate the pandemic impacts on meat demand, it can be useful to compare these WTP estimates as a percentage of February, or pre-pandemic levels. To illustrate impacts over market channel, we average percentage changes in the two beef and two pork options for broad measures of beef and pork demand adjustment. Doing so reveals retail demand in April was 4% and 9% higher than February for beef and pork, while declining 6% and 7% in food service, respectively. The larger percentage decline in food service is precisely what one would expect given state and local policies restricting restaurant service.

It should also be noted that these percentage demand-change estimates are conditional on consumers making a similar number of retail and food service purchases as in February. If a respondent was less likely to enter a restaurant, perhaps because it was closed or restricted by public health orders, then food service demand would be reduced further. Similarly, if a respondent was making more grocery-store visits, then retail demand would be higher, as these WTP changes would apply to a larger number of shopping trips. Future research, perhaps leveraging external data on changes in retail and food-service shopping-trip frequency, could further refine demand change estimates.

### 3.4. Consumer Confidence

Given the myriad of life-changing aspects of the pandemic experience, it is not surprising that household finances have been altered for many consumers. As previously indicated, early in the pandemic, consumer sentiment, as monitored by the University of Michigan’s sentiment index, fell by the most on record [28]. Coupling this with the wealth of literature indicating meat is a normal good, with beef demand being particularly sensitive to economic conditions, it is useful to leverage MDM data to examine related sentiment impacts on meat demand across households.

Accordingly, beginning in April, sentiment questions from the long-running University of Michigan Consumer Sentiment project were added. Specifically, respondents are asked, “Would you say that you (and your family living there) are better off or worse off financially than you were a year ago?” Answers provided were “Better Now”, “Same”, “Worse Now”, or “Don’t Know”. We re-estimated the multinomial logit models fit to the choice experiment data separately for consumers in each of the consumer sentiment response categories. 

As shown in Table 3 and Table 4, the beef and pork demand in both retail and food service is about 50% higher for those stating better financial conditions than those with conditions the same as last year. Given the unprecedented nature of federal aid in the CARES (Coronavirus Aid, Relief, and Economic Security) Act and the number of US households who had incomes rise or fall during the pandemic this is important to appreciate the implications for meat demand. 

There has been debate over restaurants closures and how consumers will respond when restaurants re-open more fully. Here we find substantially higher food service demand for those more confident financially. This may indirectly support the notion in broader discussions that it is not sufficient to “be open” for dine-in. Rather, until consumers are confident, feel safe, etc., their “return to normal” will be limited and food service demand may remain weakened from pre-pandemic levels until their overall financial sentiment recovers.

We can further dissect the impact of sentiment on meat demand by separately examining consumers by their pandemic employment experience and by income (above or below $100,000). Beginning in April, the MDM survey included the question, “As a result of the coronavirus pandemic, did you or someone in your family experience a change in employment status (laid off, furloughed, reduced hours, fired, etc.)?” Over the entire April-July period, 40% reported a pandemic impact on employment. 

Table 5 and Table 6 reveal how this employment impact and income combine to align with differences in meat demand across households. Consistent with the notion that meat is a normal good, meat demand is stronger in households with boosted finances and higher incomes.

Examining those reporting a pandemic employment impact (Table 5), we observe that the 7% (combined over income) who report finances have improved from the prior year and have a much stronger demand for most evaluated protein products. Of course it is natural to ask how respondents with an employment impact may have improved finances. Here, it seems likely that the historical federal stimulus effort and CARES program may have played a role. Given space constraints, we did not ask the level of CARES aide received during the April–July period.

An assessment of those not reporting a pandemic employment impact (Table 6) reveals those with incomes below $100,000 stating finances have improved from the past year (7% of respondents) have meat demand on-par or exceeding demand of those with incomes over $100,000, regardless of financial changes from the prior year. Again, while indirect, this may reflect the CARES program with stimulus funding, which would be more prevalent in households with incomes below $100,000.

Given that meat is a normal good where demand improves with consumer incomes and the above-noted differences in at-home meat availability during the pandemic, it is also useful to illustrate retail demand separately for income and at-home meat stocks subgroups [3]. As shown in Table 7, households with income below $60,000 and “normal” on-hand meat stocks were the most common group representing 36% of the May sampled population. Looking within this lower income group, 67% had normal amounts on-hand, 19% had more, and 15% had less than normal while within the higher income group 59% had normal amounts on-hand, 28% had more, & 12% had less than normal. Combined, this indicates prior to May, stock-up efforts by consumers may have been driven more by those with incomes of $60,000 and higher.

Going further and examining meat demand by subgroup, we observe those with more meat on-hand at home than normal, regardless of income, place higher value on all eight items than those with less meat on-hand at home. This likely reflects a broader set of demand differences that reflect wider heterogeneity of demand across households and perhaps a selection bias with those most desiring meat being more likely to stock up.

## 4. Discussion and Conclusions

This article uses household-level data from a recently launched continuous consumer-tracking survey, the Meat Demand Monitor. We find diverse COVID-19 pandemic impacts across US households related to at-home meat stocks, financial confidence, and income over the early months of the pandemic. These insights extend understanding of pandemic impacts on US consumers and provide a timely example of knowledge enabled by ongoing and targeted household-level data collection and analysis.

One of the unique aspects of the MDM project is the ability to provide consumer meat-demand insights in a timely manner, which is particularly important in volatile environments such as the pandemic. To provide some sense of the differences in timelines of different data products, note that on 4 August 2020 the latest MDM monthly report was posted providing demand insights for July. A little over a week later, on August 12th, the USDA ERS (Economic Research Service) released the latest retail meat price estimates for July. However, retail prices alone are insufficient to garner demand insight without associated supply information. The USDA releases quarterly per capita disappearance estimates, which are widely used to approximate domestic consumption volumes. Analysts can build their own monthly estimates of per capita disappearance [29]; however, doing so requires an estimate of meat exports and imports and as of mid-August, the latest estimates are for the month of June. Accordingly, demand indices based on USDA data have at least a 6-week lag on availability from when a month concludes. Moreover, there are practically no good data on volume of meat sales from food-away-from-home vs. food-at-home outlets. This highlights the value of timely MDM insights.Another benefit of the MDM project is it assesses particular meat products and moves away from considering all meat from a given species equal—an implicit assumption in much of existing meat demand research in the literature. As an example, consider the case of pork chop retail demand and BLS (2021) prices [30]. As shown above, retail pork-chop demand declined from April to May. Subsequently, BLS reported that US city average prices for pork chops were higher in May than April. If consumer demand was weaker and prices still rose, then a decline in supply fundamentally was at play [31]. These insights can be made on evaluated products ahead of broader insights following the USDA’s more aggregated information being available.

The National Academies of Science, Engineering, and Medicine (2019) recently published a consensus report edited and authored by multiple agricultural economists [32]. That report reviewed, assessed, and made recommendations to multiple USDA branches with an eye towards collecting data and reporting information on US agricultural production (largely farm-level) to reflect industry change over time. Moreover, this research is a direct response to a recent National Academies Panel on Consumer Food Data Systems call for new research that “collects data at regular intervals, repeats over time at an appropriate frequency, and releases data without undue delay” [33]. In 2013, Council on Food, Agricultural, and Resource Economics (C-FARE) published a report highlighting how to value federal data products [34]. This in part reflected changes in funding of data and information products. Similarly, Parcell, Tonsor, and Schroeder (2016) provided a report to the USDA, which contains a summary of how USDA reporting of meat and livestock market information has evolved over time following passage of the Livestock Mandatory Reporting (LMR) Act of 1999 [35]. It is important to note LMR passed, and with it the nature of data and information flow publically disseminated regularly, following notable livestock industry strife in the 1990s. Much of the data the cattle-beef and swine-pork industries use most heavily today results from LMR. We similarly envision additional calls for change, more data and information on the industry, etc., as the COVID-19 pandemic evolution continues. While this study is focused on monitoring meat demand in the US, the increased insights that follow from ongoing and more targeted data collection certainly apply to other countries and food sectors. 

COVID-19 has raised a host of important questions agricultural economists can help address. As noted by Lusk (2017, p. 319), “the challenge for the future is in creating the datasets we will need to answer these and emerging questions” [11]. As shown here, the newly launched MDM project has been able to facilitate multiple insights into meat-demand aspects of the ongoing pandemic. Our hope is that this serves as a successful example of how public–private efforts can align to support new, targeted data-collection efforts which, in turn, support enhanced insights that improve decision-making. Here our focus is on US meat demand yet our vision for the value in related efforts spans to other food categories and also to supply-side issues for a myriad of industries. Given ongoing changes in the funding of research and adjustments in support of ongoing data and information efforts, we hope this example encourages additional new efforts.

## Figures and Tables

**Table 1:** Maximum retail willingness-to-pay ($/lb), February to July 2020.

Retail	Ribeye Steak	Ground Beef	Pork Chop	Bacon	Chicken Breast	Plant-Based Patty	Shrimp	Beans and Rice
February-20	$16.35	$7.18	$6.11	$4.45	$7.43	$7.76	$8.94	$2.08
March-20	$15.89	$6.90	$5.74	$4.49	$7.13	$7.98	$8.55	$2.12
April-20	$16.44	$7.72	$6.33	$5.06	$7.58	$8.23	$8.98	$2.56
May-20	$15.45	$6.94	$5.87	$4.41	$6.90	$7.57	$8.82	$1.74
June-20	$15.92	$7.41	$5.71	$4.50	$7.20	$7.52	$8.22	$1.98
July-20	$16.17	$7.59	$6.38	$5.17	$7.48	$7.71	$8.80	$2.19

**Table 2:** Maximum food-service willingness-to-pay ($/meal), February to July 2020.

Food Service	Ribeye Steak	Beef Hamburger	Pork Chop	Baby Back Ribs	Chicken Breast	Plant-Based Patty	Shrimp	Salmon
February-20	$25.79	$18.88	$14.92	$17.69	$17.26	$13.31	$16.52	$18.08
March-20	$24.90	$18.39	$14.58	$17.53	$16.79	$12.83	$17.38	$17.27
April-20	$24.65	$17.58	$13.47	$17.01	$16.17	$11.57	$16.51	$17.12
May-20	$25.30	$19.09	$15.34	$18.49	$17.57	$12.76	$17.06	$17.97
June-20	$25.43	$18.69	$15.64	$18.35	$17.48	$12.63	$17.25	$18.14
July-20	$25.65	$18.40	$14.41	$17.75	$17.30	$12.52	$17.48	$17.80

**Table 3:** Retail meat demand (willingness-to-pay (WTP), $/lb), April–July 2020, by financial sentiment.

Retail Product	Currently Better off Financially	Same Financially	Currently Worse off Financially
Ribeye Steak	$21.24	$15.31	$15.15
Ground Beef	$11.70	$7.10	$6.51
Pork Chop	$9.50	$5.91	$5.35
Bacon	$7.19	$4.62	$4.46
Chicken Breast	$10.69	$7.10	$6.47
Plant-Based Patty	$10.98	$7.26	$7.55
Shrimp	$10.86	$8.73	$8.41
Beans and Rice	$3.57	$2.01	$2.01
Number of Respondents	670	2336	1073
Overall Share of Respondents	16.43%	57.27%	26.31%

**Table 4:** Food-service meat demand (WTP, $/meal), April–July 2020, by financial sentiment.

Food Service Meal Entree	Currently Better off Financially	Same Financially	Currently Worse off Financially
Ribeye Steak	$37.15	$24.10	$23.64
Beef Hamburger	$28.24	$17.54	$17.05
Pork Chop	$22.58	$14.24	$13.36
Baby Back Ribs	$25.06	$17.34	$17.23
Chicken Breast	$24.32	$16.58	$16.35
Plant-Based Patty	$16.54	$12.01	$12.39
Shrimp	$24.88	$16.46	$16.30
Salmon	$23.46	$17.55	$17.37
Number of Respondents	676	2298	1098
Overall Share of Respondents	16.60%	56.43%	26.96%

**Table 5:** Retail meat demand (WTP, $/lb), April–July 2020, by financial sentiment and household income for households with pandemic employment impact.

Retail Product	Currently Better off Financially	Same Financially	Currently Worse off Financially	Currently Better off Financially	Same Financially	Currently Worse off Financially
	Below $100k Income			$100k+ Income		
Ribeye Steak	$22.03	$18.13	$15.85	$50.46	$19.75	$16.30
Ground Beef	$13.73	$9.96	$6.83	$36.47	$11.60	$8.20
Pork Chop	$11.30	$7.99	$5.48	$32.61	$10.84	$7.42
Bacon	$8.29	$6.81	$4.69	$23.36	$9.06	$5.85
Chicken Breast	$10.52	$9.43	$6.87	$33.12	$12.17	$7.94
Plant-Based Patty	$9.68	$8.16	$8.24	$34.69	$10.20	$10.30
Shrimp	$9.75	$9.38	$8.82	$30.30	$11.98	$9.67
Beans and Rice	$3.40	$3.56	$2.40	$19.09	$3.56	$3.68
Number of Respondents	224	517	577	98	133	76
Overall Share of Respondents	5%	13%	14%	2%	3%	2%

**Table 6:** Retail meat demand (WTP, $/lb), April–July 2020, by financial sentiment and household income for households without a pandemic employment impact.

Retail Product	Currently Better off Financially	Same Financially	Currently Worse off Financially	Currently Better off Financially	Same Financially	Currently Worse off Financially
	Below $100k Income			$100k+ Income		
Ribeye Steak	$17.34	$13.93	$13.18	$17.20	$15.63	$16.93
Ground Beef	$8.43	$6.09	$5.56	$7.32	$6.74	$7.03
Pork Chop	$6.24	$5.00	$4.31	$6.40	$5.85	$6.63
Bacon	$5.61	$3.83	$3.99	$4.84	$4.28	$3.92
Chicken Breast	$8.11	$6.04	$5.20	$8.23	$7.19	$8.20
Plant-Based Patty	$8.13	$7.00	$5.70	$8.18	$7.08	$5.16
Shrimp	$9.55	$7.64	$7.05	$8.84	$10.26	$10.2
Beans and Rice	$2.45	$1.71	$1.11	$1.90	$1.72	$2.07
Number of Respondents	273	1392	360	74	293	60
Overall Share of Respondents	7%	34%	9%	2%	7%	1%

**Table 7:** Retail meat demand (WTP, $/lb), by household income and at-home meat supplies, May 2020.

	Income below $60,000			Income of $60,000 or Higher		
Retail Product	More Meat On-Hand than Normal	Same Amount as Normal	Less Meat On-Hand than Normal	More Meat On-Hand Than Normal	Same Amount as Normal	Less Meat On-Hand than Normal
Ribeye Steak	$18.11	$14.64	$13.43	$19.26	$15.27	$15.09
Ground Beef	$10.17	$6.94	$4.51	$9.94	$6.50	$6.52
Pork Chop	$8.21	$5.68	$4.07	$8.78	$5.71	$5.78
Bacon	$6.97	$4.83	$2.7	$6.88	$3.79	$2.75
Chicken Breast	$9.36	$6.68	$4.35	$9.87	$6.92	$6.34
Plant-Based Patty	$9.85	$7.10	$5.28	$11.74	$6.05	$8.89
Shrimp	$9.68	$8.24	$7.40	$11.25	$9.22	$9.01
Beans and Rice	$2.37	$2.39	$0.13	$3.72	$0.63	$2.40
Number of Respondents	104	372	8	133	278	58
Overall Share of Respondents	10.14%	36.26%	7.89%	12.96%	27.10%	5.65%
Conditional Share of Respondents, Income below $60,000	18.67%	66.79%	14.54%			
Conditional Share of Respondents, Income of $60,000 or Higher				28.36%	59.28%	12.37%

## Data Availability

Data utilized here are available online at https://www.agmanager.info/livestock-meat/meat-demand/monthly-meat-demand-monitor-survey-data (accessed on 5 April 2021). Any users are encouraged to review the Project Methodology document and follow the described data-quality filters.
